# Adiponectin 1 receptor is increased but not adiponectin levels in the tumour microenvironment of postmenopausal women with breast cancer

**DOI:** 10.1007/s11033-025-11392-4

**Published:** 2025-12-28

**Authors:** Diego García-Mata, Alberto Tenorio-Torres, Ramón Mauricio Coral-Vázquez, Alexandra Dávalos-Herrera, Verónica Bautista-Piña, Patricia Canto

**Affiliations:** 1 Unidad de Investigación en Obesidad, Facultad de Medicina, Universidad Nacional Autónoma de México, Ciudad de México, México; 2 Subdirección de Investigación Clínica, Dirección de Investigación, Instituto Nacional de Ciencias Médicas y Nutrición “Salvador Zubirán”, Ciudad de México, México; 3 Instituto de Enfermedades de la Mama, FUCAM, Ciudad de México, México; 4 Servicio de Oncología Quirúrgica,, Centro Médico Nacional “20 de Noviembre”, Instituto de Seguridad y Servicios Sociales de los Trabajadores del Estado, Ciudad de México, México; 5 Sección de Posgrado e Investigación, Escuela Superior de Medicina, Instituto Politécnico Nacional, Ciudad de México, México; 6 Subdirección de Enseñanza e Investigación, Centro Médico Nacional “20 de Noviembre”, Instituto de Seguridad y Servicios Sociales de los Trabajadores del Estado, Ciudad de México, México

**Keywords:** Adiponectin, ADIPOR1, Microenvironment of breast cancer, postmenopausal Mexican-Mestizo women, normal weight, Obesity

## Abstract

**Background:**

To analyze the ADIPOQ and ADIPOR1 levels in breast tumour tissue and adjacent adipose tissue of postmenopausal women with this cancer. We hypothesized that the tumour microenvironment (TME) of the breast had lower levels of ADIPOQ and ADIPOR1 in postmenopausal women with obesity than in those with a normal BMI.

**Methods and results:**

Twenty women with normal body mass index (BMI) and 20 with obesity, all of them postmenopausal and with breast cancer (BC) were included. We obtained during surgery fresh breast tumour tissue and a fragment of breast adipose tissue adjacent to the tumour and analyzed the levels of adiponectin (ADIPOQ) and its receptor ADIPOR1 by Western blot. Statistical power of the study was > 80% with a *p* < 0.05ADIPOR1 protein levels were higher in breast tumour tissue *versus* breast adipose tissue adjacent to the tumour in postmenopausal women with normal BMI and postmenopausal women with obesity (*p* = 0.0012 and *p* = 0.0001, respectively). Moreover, we observed higher ADIPOR1 levels only in breast adipose tissue adjacent to the tumour in postmenopausal women with obesity and tumour size > 2.0 cm and clinical stage II/III (*p* = 0.019 and *p* = 0.025, respectively) versus postmenopausal women with a normal BMI. We did not observe differences in ADIPOQ.

**Conclusions:**

ADIPOR1 levels were higher in breast tumour tissue compared to breast adipose tissue adjacent to the tumour in both postmenopausal women with normal BMI or with obesity. Besides, ADIPOR1 levels were higher in breast adipose tissue adjacent to the tumour of postmenopausal women and obesity, with a more aggressive breast tumour.

**Supplementary Information:**

The online version contains supplementary material available at 10.1007/s11033-025-11392-4.

## Introduction

Breast cancer (BC) is one of the most common cancers in the world [1], and Mexico is not the exception, being the first cause of death from cancer in women; only for 2022 were recorded 23,790 new cases of BC among the population of 20 years old and over, being the national incidence of this disease of 27.64 cases per 100,000 inhabitants for that same year (https://www.inegi.org.mx › EAP_CMAMA239; accessed May 13, 2025). The average age overall at diagnosis of breast cancer in women is 62 years [2]; however, for Mexico, the average age of diagnosis is 58.2 years (https://www.gob.mx/salud/documentos/epidemiologia-del-cancer-de-mama; accessed May 13, 2025).

Several modifiable and non-modifiable risk factors participate in the etiology of BC [3, 4], including obesity, an important modifiable risk factor for postmenopausal breast cancer [5, 6]. In Mexico, obesity is considered a major public health problem, since the National Survey on Health and Ageing (ENASEM) 2021, reported that the prevalence of this disease in women aged 50 years or older was 30.3% (https://www.inegi.org.mx/programas/enasem/2021; accessed May 14, 2025).

Obesity in postmenopausal women is a risk factor for the development of estrogen receptor positive (ERα)/luminal BC subtypes [7]; it is associated with a worse prognosis, worse disease-free survival and overall survival in comparison to women of normal body mass index (BMI) [7, 8].

Since mammary epithelial cells are interspersed within adipose tissue, this direct interaction occurs through a functional paracrine mechanism [9, 10]. Further, it has been described in BC that components of the tumour microenvironment (TME), including adipocytes, are a predominant cellular component of the TME, play a central role in the biological behaviour of this cancer [11–13].

Several studies have demonstrated that adipocytes exhibit a dysfunctional adipokine secretion profile in BC, including adiponectin (ADIPOQ) [14–16]. This adipokine acts by binding to adiponectin receptor 1 (ADIPOR1) and adiponectin receptor 2 (ADIPOR2). Although both are expressed in breast tumour tissue [17, 18], ADIPOR1 levels are higher than those of ADIPOR2 [18].

This adipokine can inhibit cell proliferation and promote apoptosis [19, 20], which could protect against breast carcinogenesis and progression; however, it has been suggested that ADIPOQ participates in BC growth through the presence of the estrogen receptor α (ERα) in the tumour [16, 21].

Since our research group demonstrated by immunohistochemistry, that women with overweight or obesity had a lower expression of this adipokine and higher ADIPOR1 expression in breast cancer tissue [18], and that adiponectin plays a role in the TME of breast cancer; the objective of this study is to analyze the ADIPOQ and ADIPOR1 levels in fresh breast tumour tissue and adjacent adipose tissue of postmenopausal women with this cancer.

We hypothesized that breast tumor tissue has higher ADIPOQ and ADIPOR1 levels compared to adjacent adipose tissue in postmenopausal women with obesity *versu*s those with a normal BMI.

## Subjects and methods

### Subjects

The Research and Research Ethics Committees of the Instituto de Enfermedades de la Mama (FUCAM), Mexico (CEI/PI-19-11/2020 prospectively registered trial) and of the Facultad de Medicina, Universidad Nacional Autónoma de Mexico (registration number FMED/CEI/RMWC/015/2020) approved the study, and informed consent was obtained from all participants.

This is a cross-sectional study in which forty postmenopausal women with BC diagnosis were included from the Instituto de Enfermedades de la Mama (FUCAM), Mexico, between 2020 and 2021, and were grouped according to their body mass index (BMI) as normal (20–24.9 kg/m^2^) or with obesity (BMI ≥ 30.0 kg/m^2^). The study design followed the STROBE guidelines.

General, clinical, and variables associated with BC, as well as histopathological characteristics, were retrospectively reviewed in patients’ medical records. The inclusion criteria were that women had a histopathological diagnosis of early-stage operable invasive breast cancer and had luminal BC subtypes. Exclusion criteria were women with a family history of breast cancer, with a history of other previous neoplasias, women with metastatic carcinoma or who had received prior treatment for the current pathology (i.e. tamoxifen use, radiotherapy and/or chemotherapy, or received neoadjuvant chemotherapy).

## Methods

### Tissue samples

During surgery, following tumour resection, around 200 to 600 mg of fresh breast tumour tissue and a fragment of fresh breast adipose tissue adjacent to the tumour were selected and collected by a pathologist from all postmenopausal women and kept at − 80 °C.

### Western blotting

Proteins of interest were analyzed by Western blot. For this purpose, approximately 150 mg of each tissue was homogenized in RIPA buffer (Santa Cruz Biotechnology, Inc., Santa Cruz, California, USA) containing protease and phosphatase inhibitors (P2714 and P2850, Sigma-Aldrich, St. Louis, Missouri, USA), along with the addition of 5 mM Na3VO4 and 3 mM NaF (Santa Cruz Biotechnology, Inc., Santa Cruz, California, USA). Afterwards, 50–80 µg of protein was loaded onto a denaturing SDS-PAGE gel and electrotransferred to a nitrocellulose membrane (0.45 μm, P01-88018, Thermo Fisher Scientific Inc., Waltham, Massachusetts, USA). As primary antibodies, we used anti-Adiponectin (MA1-054; dilution 1:500) (Invitrogen, Thermo Fisher Scientific Inc., Waltham, Massachusetts, USA) and anti-AdipoR1 (sc-518030; dilution 1:100) (Santa Cruz Biotechnology, Inc., Santa Cruz, California, USA). Additionally, we used anti-alpha-tubulin (sc-8035; dilution 1:500) (Santa Cruz Biotechnology, Inc., Santa Cruz, California, USA) as a loading control. The secondary antibody was Peroxidase AffiniPure^®^ Donkey Anti-Mouse IgG (715-035-150; dilution 1:4000) (Jackson ImmunoResearch Laboratories Inc., West Baltimore Pike, West Grove, PA, USA). The interaction between the antigen and antibody was assessed using the SuperSignal West Femto Chemiluminescent Substrate kit (34095, Thermo Scientific Inc., Waltham, Massachusetts, USA). Band intensities were digitally quantified using LI-COR Image Studio software (http://www.licor.com/bio/image_studio) (LI-COR Biosciences, Lincoln, New England, USA). Images of Western blot standardization for anti-Adiponectin and anti-AdipoR1 are included as supplementary Figs. 1 and 2, respectively.

### Statistical analysis

Data for quantitative variables are presented as ranges, and for qualitative variables as absolute and relative frequencies. Comparisons between continuous numerical variables were performed using the Mann-Whitney test or the unpaired t-test, while Fisher’s exact test was used for qualitative variables. Protein levels were analyzed using the Wilcoxon test or the paired t-test, depending on their distribution.

Sample size and power were calculated to achieve 80% power and an alpha risk of 0.05, assuming a population effect size of 0.6, using a two-tailed two-sample t test, following the study by Körner et al., [11]. All analyses were performed using SPSS v21.0 (IBM SPSS Software, Armonk, NY) and GraphPad Prism v6 (GraphPad Software, San Diego, CA). A p-value < 0.05 was accepted as statistically significant.

## Results

The general and histological characteristics of all women are presented in Table 1. Twenty postmenopausal women with normal BMI and twenty with obesity, all of them with breast cancer, were studied. The age range is 50-to-89 for women with normal BMI and 49-to-79 for women with obesity. One woman with normal BMI had molecular subtype luminal B, while the rest of the women had molecular subtype luminal A; besides, only one woman with obesity presented clinical stage III.

**Table 1:** General and histological characteristics of postmenopausal women with breast cancer and normal BMI or with obesity

Variables	Women with normal BMI (*n* = 20)	Women with obesity (*n* = 20)	*p*
Age (years)*	50–89	49–77	0.7631
BMI (kg/m^2^)*	18.2–24.9	30.1–46.9	0.0001
Menopause (years)*	45–55	45–56	0.1894
Tumour size, n (%)			0.1110
≤2 cm	12 (60.0)	6 (30.0)	
>2 cm	8 (40.0)	14 (70.0)	
Clinical stage, *n* (%)			0.4506
I	6 (30.0)	3 (15.0)	
II/III	14 (70.0)	17 (85.0)	
Ki67, n (%)			0.3008
≤20%	12 (60.0)	16 (80.0)	
>20%	8 (40.0)	4 (20.0)	
HER, n (%)			1.0000
Positive	1 (5.0)	0 (0.0)	
Negative	19 (95.0)	20 (100)	
Surgery type, n (%)			
Quadrantectomy	3 (15.0)	5 (25.0)	0.6948
Mastectomy	17 (85.0)	15 (75.0)	

BMI = Body mass index; n = number of women; *values are in range

We did not observe differences in reproductive history, intake of oral contraceptives, estrogen replacement therapy, smoking habits, presence of diabetes, and use of metformin (data not shown).

**Fig. 1:**
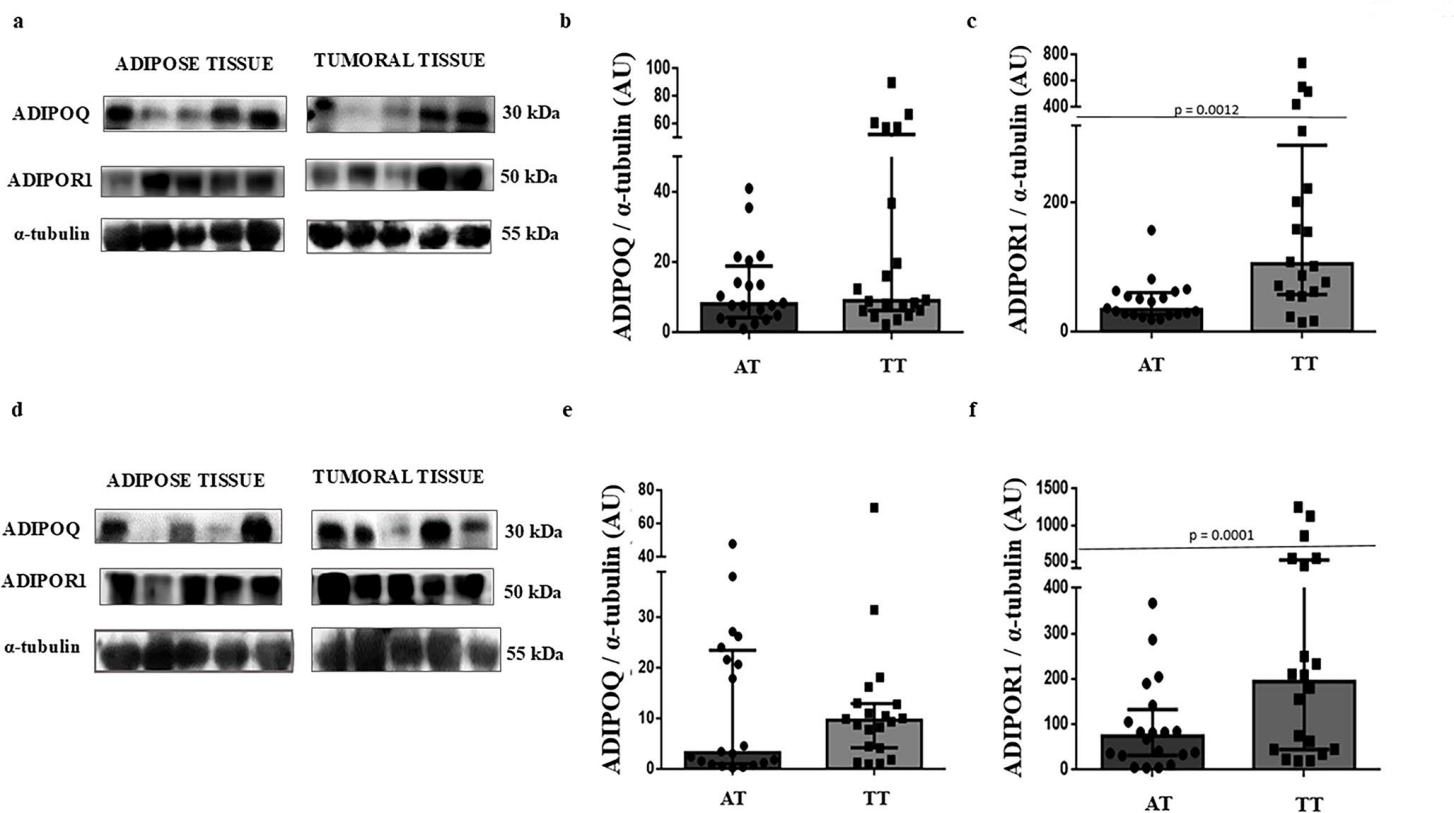
Protein levels of adiponectin and its receptor in breast tumour tissue and breast adipose tissue adjacent to the tumour in postmenopausal women with normal BMI (a-to-c) or with obesity (d-to-f). a and b, Representative results from Western Blot analysis of protein levels of ADIPOQ and ADIPOR1. b and e, Graphical densitometry analysis shows no changes in ADIPOQ. c and f, ADIPOR1 levels are increased in TT compared to AT (*p* = 0.001) for both, postmenopausal women with normal BMI of with obesity. Data are expressed as median with interquartile range *N* = 20 postmenopausal women with normal BMI and 20 postmenopausal women with obesity. ADIPOQ = adiponectin; ADIPOR1 = ADIPOQ receptor; BMI = body mass index; AT = breast adipose tissue adjacent to the tumour; TT = breast tumoural tissue

The ADIPOQ protein levels in breast tumour tissue and adjacent breast adipose tissue were similar in both postmenopausal women with normal BMI and those with obesity (Fig. 1, Panels a, b, d, and e).

In contrast, we found that ADIPOR1 protein levels were higher in breast tumour tissue versus breast adipose tissue adjacent to the tumour, with a 3-fold increase for postmenopausal women with normal BMI and a 2.5-fold increase for postmenopausal women with obesity, and these differences were significant (*p* = 0.0012 and *p* = 0.0001, respectively; Fig. 1, Panels a, c, d, and f).

**Fig. 2:**
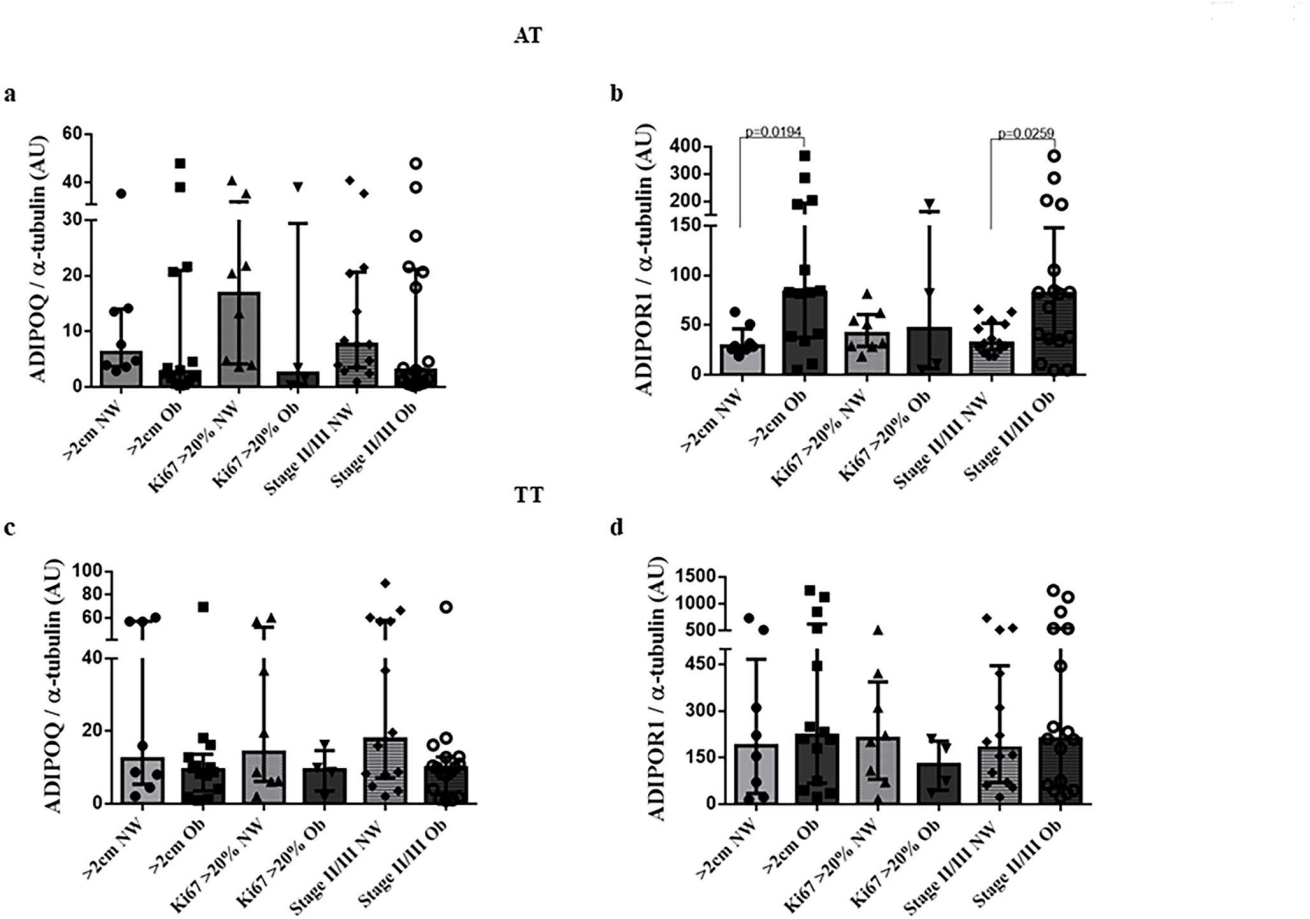
Protein levels of adiponectin and its receptor in breast adipose tissue adjacent to the tumour (a and b) and in breast tumour tissue (c and d) in postmenopausal women with normal BMI or with obesity and their relationship with the aggressiveness breast cancer. b. The ADIPOR1 levels were significantly higher in breast adipose tissue adjacent to the tumour of postmenopausal women with obesity with tumour ≥ 2 cm and II/III clinical stage in comparison to those with normal BMI (*p* < 0.0194 and *p* < 0.0259, respectively) *N* = 20 postmenopausal women with normal BMI and 20 postmenopausal women with obesity. ADIPOQ = adiponectin; ADIPOR1 = ADIPOQ receptor; AT = breast adipose tissue adjacent to the tumour; TT = breast tumoural tissue. NW = postmenopausal women with normal weight; Ob = postmenopausal women with obesity

On the other hand, we observed significantly higher ADIPOR1 levels only in breast adipose tissue adjacent to the tumour in postmenopausal women with obesity and tumour size > 2.0 cm and clinical stage II/III (*p* = 0.019 and *p* = 0.025, respectively, Fig. 2, Panel b) compared with postmenopausal women with a normal BMI. Although we did not find differences in ADIPOQ levels in both tissues (Fig. 2, Panels a and c) or in ADIPOR1 levels in breast tumours (Fig. 2, Panel d).

Table 2 shows ADIPOQ and ADIPOR1 levels in both tissues of postmenopausal women with normal BMI or obesity.

**Table 2:** ADIPOQ andADIPOR1 levels in breast tumour tissue and breast adipose tissue adjacent to the tumor of postmenopausal women with normal BMI or with obesity

	Women with normal BMI (*n* = 20)	Women with obesity (*n* = 20)
*Protein levels in AT*		
ADIPOQ	8.0 (2.4–35.5)	3.2 (0.25–47.9)
ADIPOR1	34.1 (18.6–157.7)	74.9 (4.5–367.3)
*Protein levels in TT*		
ADIPOQ	8.9 (2.1–89.9)	9.6 (0.93–69.5)
ADIPOR1	105.2 (14.5–736.3)	194.6 (19.2–1251.0)

BMI = Body mass index; n = number of women; AT = breast adipose tissue adjacent to the tumor; TT = breast tumor tissue; ADIPOQ = adiponectin; ADIPOR1 = receptor of adiponectin. Values are in median (ranges)

## Discussion

Obesity in women with breast cancer is associated with a decrease in disease-free survival, relapse-free survival, breast cancer-specific survival and overall survival [22]. In postmenopausal women, approximately 70% to 80% of all breast cancers are ERα [23]. The latter is important because it has been reported that, among postmenopausal women with this cancer and obesity, the most frequent molecular subtype is luminal A (ERα + and/or PR+, HER2+, and Ki-67 low) [24, 25], as we observed in our study.

In the mammary gland, the adipose tissue occupies around to 7-to-56% of the fat volume of the total gland volume, with 3.6-to-37.6% of the weight of this tissue of the total breast weight [26, 27]. In an obese state, the augmented adiposity in the mammary gland is associated with adipocyte hypertrophy, which promotes the presence of hypoxia, nutrient deprivation, metabolic stress, and an inflammatory milieu [28], creating a favourable local environment for the development of breast cancer.

Mammary gland adipose tissue secretes several adipokines, and due to the close relationship between breast cancer cells and adjacent adipose tissue, it could lead to the reprogramming of the TME, and therefore, altered secretion of adipokines, including adiponectin [29]. Regarding this adipokine, it has been described to have an anticancer action via several mechanisms [19, 30, 31], acting by binding to its adiponectin receptors (ADIPOR1 and ADIPOR2), thereby triggering their intracellular signalling cascades. Furthermore, it has been described that ADIPOQ and their receptor are expressed in the TME of breast cancer [14]. Therefore, in the present study, we analyzed the ADIPOQ and ADIPOR1 protein levels in fresh breast tumour tissue and fresh breast adipose tissue adjacent to the tumour of postmenopausal women with normal BMI or with obesity, who had a histopathological diagnosis of early-stage operable invasive breast cancer and had a predominant luminal A subtype. To our knowledge, this constitutes the first study to investigate possible differences in ADIPOQ and ADIPOR1 protein levels in both tissues among this group of women with normal BMI or obesity.

Most previous studies by other groups and our own [18] that analyzed ADIPOQ and/or ADIPOR1 levels were performed on paraffin-embedded breast tumor tissue using immunohistochemistry. Therefore, it is difficult to compare the results of this study with those of other studies published in the literature.

Körner et al. [11] performed a study in which analyzed the expression of ADIPOQ and ADIPOR1 genes in breast tumour tissue and adipose tissue adjacent to the tumour of women with this cancer, the authors found that ADIPOQ mRNA was more highly expressed in adjacent adipose tissue than in cancerous breast tissue in comparison to women without cancer. Further, ADIPOR1 mRNA expression was observed in both tissues, but was higher in tumour tissues from women with BC than in those without this cancer. However, it is important to mention that women with breast cancer were overweight or had obesity (the same as their controls), and they did not compare whether the expression was different between women with BC and normal BMI *versus* women with obesity.

Moreover, Fletcher et al. [32] conducted an in vitro study in which they observed, in epithelial cell lines, an increase in AdipoR1 and a decrease in adiponectin expression in human breast cancer adipose tissue explants.

Contrary to the above studies, we investigated ADIPOQ and ADIPOR1 at the protein level and found that ADIPOQ levels did not differ between the two tissues in women with normal BMI or those with obesity. Similar to Körner et al. [11], we found that ADIPOR1 levels were higher in breast tumour tissue than in adjacent adipose tissue, in both postmenopausal women with normal BMI and those with obesity.

Increased ADIPOR1 protein levels may serve as a compensatory mechanism to offset the low adiponectin levels [14]. Although this phenomenon was only present in breast tumour tissue and not in the adjacent breast adipose tissue of both postmenopausal women with normal BMI or with obesity, we hypothesized that it could be due to the autocrine effect of ADIPOR1 on breast tumour tissue, reducing tumour development or progression; however, is necessary to perform functional assays in order to probe this hypothesis.

On the other hand, Llanos et al. [15] conducted an investigation analyzing the gene expression of several adipokines, including ADIPOQ and ADIPOR1, in the breast TME and their association with tumour clinicopathological characteristics. The authors reported that a lower ADIPOQ expression was associated with more aggressive phenotypes of breast cancer, and higher ADIPOR1 expression was associated only with larger tumours. However, these authors included pre- and postmenopausal women; most participants were of African descent, and several molecular subtypes of breast cancer were included. Furthermore, they did not analyze ADIPOQ or ADIPOR1.

Interestingly, our results show that significantly higher ADIPOR1 levels were associated with a more aggressive breast tumour only in postmenopausal women with obesity. We suggested that this phenomenon could be because the paracrine effect of ADIPOR1 in the breast adipose tissue adjacent to the tumour was not sufficient to reduce tumour progression, and that this could be one of the reasons why postmenopausal women with obesity develop more aggressive breast cancer [22].

The possible mechanism proposed for this action is that upon binding of ADIPOQ to ADIPOR1, the adaptor protein, phosphotyrosine interacting with PH domain and leucine zipper 1 (APPL1) recruitment is induced, leading to the activation of 5’-adenosine monophosphate-activated protein kinase (AMPK), thereby inhibiting downstream signaling pathways of key kinases controlling cell proliferation, survival and apoptosis [33].

A limitation of this study was that using a cross-sectional design makes it difficult to determine the possible causal effects of our findings; furthermore, we did not perform functional assays to investigate the possible mechanisms involved in the effect that higher levels of ADIPOR1 have on the tumor microenvironment. Despite these limitations, the strengths of the study are that all women were postmenopausal and only included women with early-stage cancer and that we were statistically powered to find significant results.

## Conclusions

We did not observe any differences in ADIPOQ protein levels in breast tumour tissue or adjacent breast adipose tissue. However, we found that ADIPOR1 protein levels were higher in breast tumour tissue than in adjacent breast adipose tissue in both postmenopausal women with normal BMI and those with obesity. Besides, ADIPOR1 protein levels were higher in breast adipose tissue adjacent to tumours in postmenopausal women and in obese women with more aggressive breast tumours.

## Supplementary Information

Below is the link to the electronic supplementary material.

Supplementary Figure 1. Adiponectin (ADIPOQ) detection (30kDa) in breast tumoral tissue and adipose tissue of women with breast cancer

Supplementary Figure 2. Adiponectin receptor (ADIPOR1) detection (50kDa) in breast tumoral tissue and adipose tissue of women with breast cancer.

## Data Availability

No datasets were generated or analysed during the current study.

## Abbreviations

ADIPOQ: Adiponectin
ADIPOR1: Adiponectin receptor 1
ADIPOR2: Adiponectin receptor 2
BC: Breast cancer
BMI: Body mass index
ERα: Estrogen receptor alpha
PR: Progesterone receptor
TME: Tumour microenvironment
