# Supplementary figures and images for: Adiponectin 1 receptor is increased but not adiponectin levels in the tumour microenvironment of postmenopausal women with breast cancer
Source: Mol Biol Rep. 2025 Dec 28;53(1):220. doi: 10.1007/s11033-025-11392-4 (PMC12745321; doi:10.1007/s11033-025-11392-4)

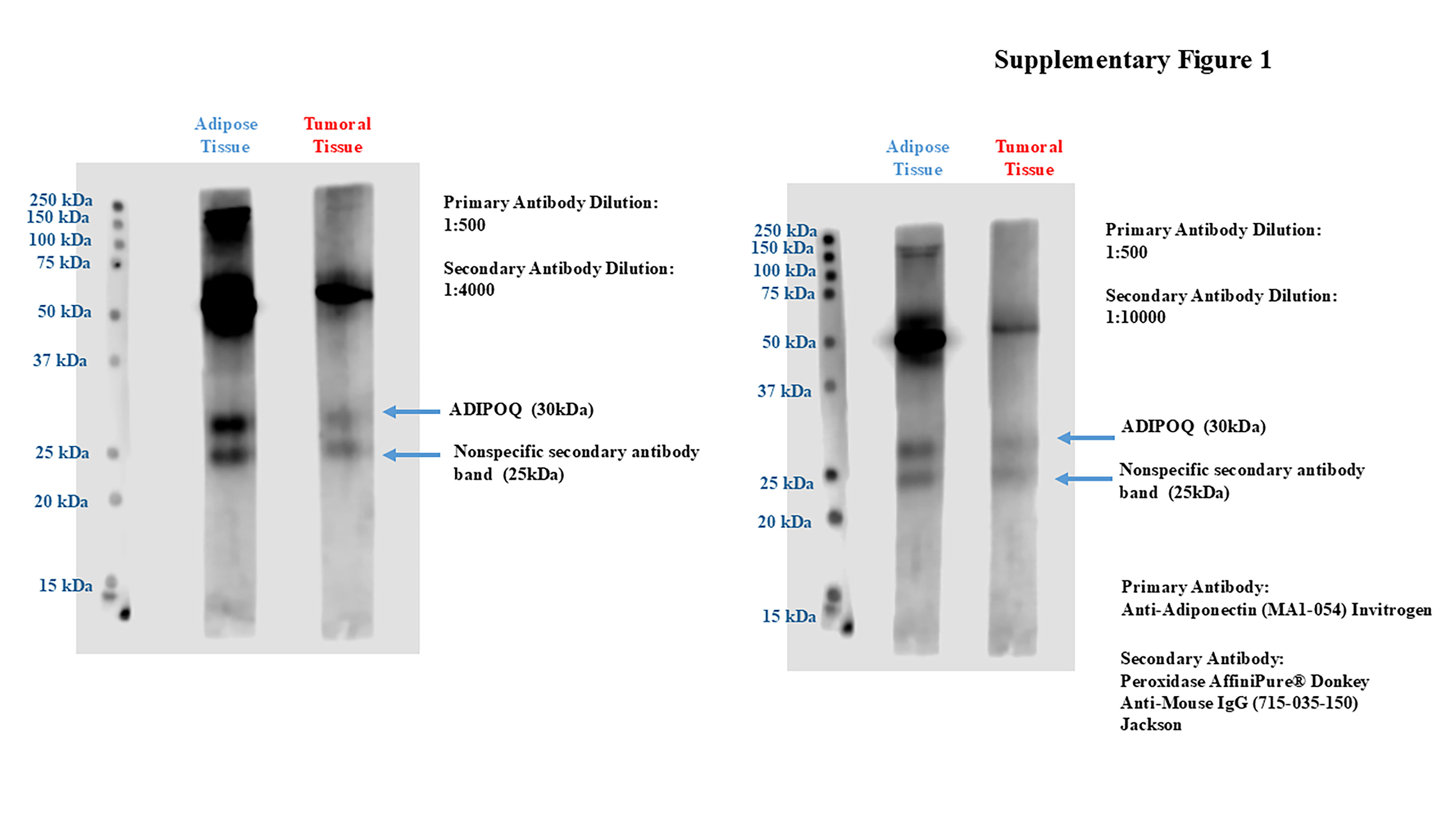

Supplement: Supplementary file 1 — Supplementary Figure 1. Adiponectin (ADIPOQ) detection (30kDa) in breast tumoral tissue and adipose tissue of women with breast cancer [file 11033_2025_11392_MOESM1_ESM.jpg]

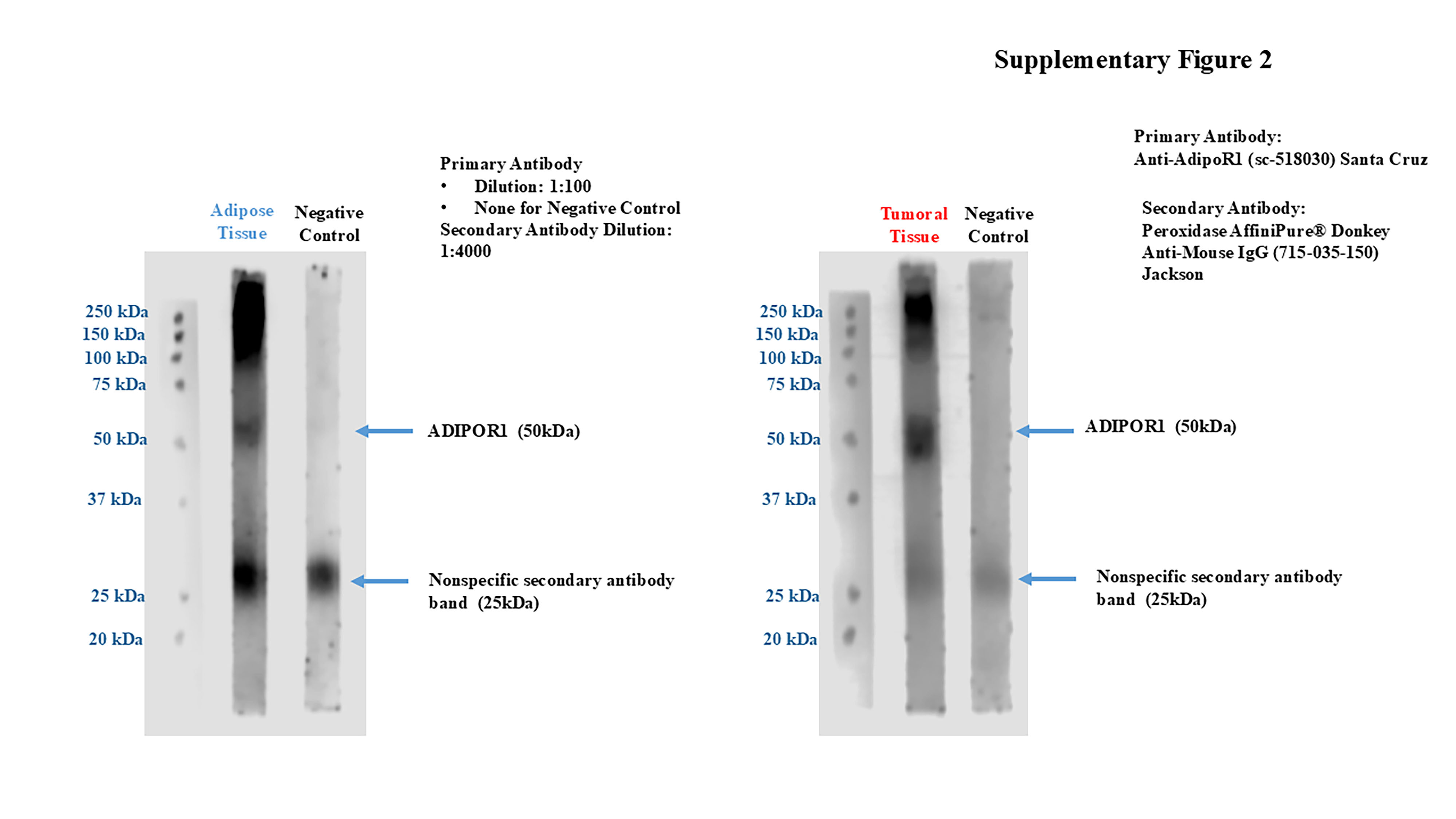

Supplement: Supplementary file 2 — Supplementary Figure 2. Adiponectin receptor (ADIPOR1) detection (50kDa) in breast tumoral tissue and adipose tissue of women with breast cancer. [file 11033_2025_11392_MOESM2_ESM.jpg]
